# Analyzing long-term water quality of lakes in Rhode Island and the northeastern United States with an anomaly approach

**DOI:** 10.1002/ecs2.3555

**Published:** 2021-06-09

**Authors:** J. W. Hollister, D. Q. Kellogg, B. J. Kreakie, S. D. Shivers, W. B. Milstead, E. M. Herron, L. T. Green, A. J. Gold

**Affiliations:** 1 U.S. Environmental Protection Agency, Office of Research and Development, Atlantic Coastal Environmental Sciences Division, Narragansett, Rhode Island 02882 USA; 2 Department of Natural Resources Science, University of Rhode Island, Kingston, Rhode Island 02881 USA

**Keywords:** anomaly, chlorophyll, community science, lake temperature, lakes, long term, nutrients, trend analysis, volunteer monitoring

## Abstract

Addressing anthropogenic impacts on aquatic ecosystems is a focus of lake management. Controlling phosphorus and nitrogen can mitigate these impacts, but determining management effectiveness requires long-term datasets. Recent analysis of the LAke multi-scaled GeOSpatial and temporal database for the Northeast (LAGOS-NE) United States found stable water quality in the northeastern and midwestern United States; however, sub-regional trends may be obscured. We used the University of Rhode Island’s Watershed Watch Volunteer Monitoring Program (URIWW) dataset to determine if there were sub-regional (i.e., 3000 km^2^) water quality trends. URIWW has collected water quality data on Rhode Island lakes and reservoirs for over 25 yr. The LAGOS-NE and URIWW datasets allowed for comparison of water quality trends at regional and sub-regional scales, respectively. We assessed regional (LAGOS-NE) and sub-regional (URIWW) trends with yearly median anomalies calculated on a per-station basis. Sub-regionally, temperature and chlorophyll *a* increased from 1993 to 2016. Total nitrogen, total phosphorus, and the nitrogen:phosphorus ratio (N:P) were stable. At the regional scale, the LAGOS-NE dataset showed similar trends to prior studies of the LAGOS-NE with chlorophyll *a*, total nitrogen, and N:P all stable over time. Total phosphorus did show a very slight increase. In short, algal biomass, as measured by chlorophyll *a* in Rhode Island lakes and reservoirs increased, despite stability in total nitrogen, total phosphorus, and the nitrogen to phosphorus ratio. Additionally, we demonstrated both the value of long-term monitoring programs, like URIWW, for identifying trends in environmental condition, and the utility of site-specific anomalies for analyzing for long-term water quality trends.

## Introduction

Aquatic ecosystems have been altered as the result of human activities modifying nutrient cycling on a global scale (Vitousek et al. 1997, Filippelli 2008, Finlay et al. 2013). Because of their position in the landscape, lakes can function as integrators and sentinels for these anthropogenic effects (Williamson et al. 2008, Schindler 2009). Increasing nutrient inputs, particularly of nitrogen (N) and phosphorus (P), derived from intensive agriculture and densely populated urban areas have contributed to the eutrophication of many lakes (Carpenter et al. 1998, Smith 2003). This eutrophication often leads to an increase in the frequency and severity of harmful algal blooms, greater risks for human and animal health, and potential economic costs associated with eutrophic waters (Dodds et al. 2009, Paerl and Huisman 2009, Kosten et al. 2012, Michalak et al. 2013, Taranu et al. 2015, Brooks et al. 2016). To address these problems, management strategies have historically focused on reducing P inputs to lakes, but research also suggests that reducing N inputs may be more effective in certain situations (Schindler et al. 2008, Paerl et al. 2016). These studies indicate that relationships between N, P, and chlorophyll *a* exist and these relationships are spatially and temporally complex. Thus, long-term data are needed to identify trends at local, regional, and national scales.

Lake datasets that cover longer time periods and broader spatial scales are now becoming available. Programs such as the U.S. Environmental Protection Agency’s National Lakes Assessment (NLA) provide data that allow for continental-scale water quality analysis. These data allow for analyses that can be useful for managing water resources by developing water quality criteria for N, P, and chlorophyll *a* (Herlihy et al. 2013, Yuan et al. 2014). Studying temporal trends across large spatial scales can illustrate the effects of eutrophication such as the degradation of oligotrophic systems as P increases (Stoddard et al. 2016). Broad-scale data can also be used for water quality modeling across a range of spatial scales including for predicting lake trophic state, which is indicative of ecosystem condition (Hollister et al. 2016, Nojavan et al. 2019). These trophic state models indicate that landscape variables (e.g., ecoregion, elevation, and latitude) are important and that regional trends exist. Lake-specific drivers have also been shown to be important for predicting continental-scale water quality which adds an additional layer of complexity (Read et al. 2015). Despite these challenges, it is important to study lakes at multiple spatial scales because emergent trends on regional or continental scales may or may not be present in individual lakes (Cheruvelil et al. 2013, Lottig et al. 2014).

Previous studies using regional data from the northeastern and midwestern United States approximately 400,000 km^2^ and 1,000,000 km^2^ respectively, have investigated spatial and temporal water quality trends and have demonstrated that drivers explaining changes spatially may not explain changes temporally. These differences may be due to complex interactions occurring at the different scales through time (Lottig et al. 2017). Additionally, differences between regions can cause nutrient (N and P) trends to have different drivers compared to ratios of the nutrients, and these may or may not align with sub-continental (i.e., ~1,800,000 km^2^) trends (Collins et al. 2017). Similarly, trends of N, P, and chlorophyll *a* differ as factors such as land use and climate vary between regions, particularly when comparing the northeastern and midwestern U.S. regions (Filstrup et al. 2014, 2018). Furthermore, little change in nutrients and chlorophyll *a* was reported over a 25-yr period for these regions (Oliver et al. 2017). Given what is known about long-term trends in water quality within the broader regions of the northeastern and midwestern United States, we were curious if the lack of trends was also present in water quality at a sub-regional scale, using data on the 3000 km^2^ area that encompasses a number of Rhode Island lakes and reservoirs.

In this study, we focus on the state of Rhode Island for several reasons. Rhode Island has the second highest population density in the country, yet still has a mix of land use/land cover with developed areas making up ~21% of the state, forested areas ~37%, wetlands ~11%, and agriculture ~3%. The remainder of the land area is made up other land-use/land-cover classes. Given these facts, Rhode Island lakes occur across a gradient of land use/land cover and face a suite of human pressures that most lakes in the nation are also facing with nutrients and pathogens being of particular concern in the state (Rhode Island Division of Planning 2016). Lastly, Rhode Island provides an ideal study area as significant, long-term datasets are available for lakes making it possible to examine water quality trends.

The goals of this study were to examine ~25 yr of lake and reservoir data in Rhode Island and answer two questions. First, are there state-wide trends in total nitrogen (TN), total phosphorus (TP), total nitrogen-to-total phosphorus ratio (TN:TP), chlorophyll *a*, and lake temperature? Second, are water quality trends in Rhode Island similar to regional trends in the northeastern United States? Another objective of this paper was to apply existing methods for examining long-term climate records (e.g., Jones and Hulme 1996) to water quality data in order to examine long-term trends. We conducted this analysis using open data from the URI Watershed Watch program and the LAke multi-scaled GeOSpatial and temporal database for the Northeast (LAGOS-NE) project and the analysis in its entirety is available for independent reproduction at https://github.com/usepa/ri_wq_trends and is archived at https://doi.org/10.5281/zenodo.4306765 (Soranno et al. 2017, 2019, Hollister et al. 2020, Stachelek and Oliver 2020).

## Methods

### Study area and data

**Fig. 1.**
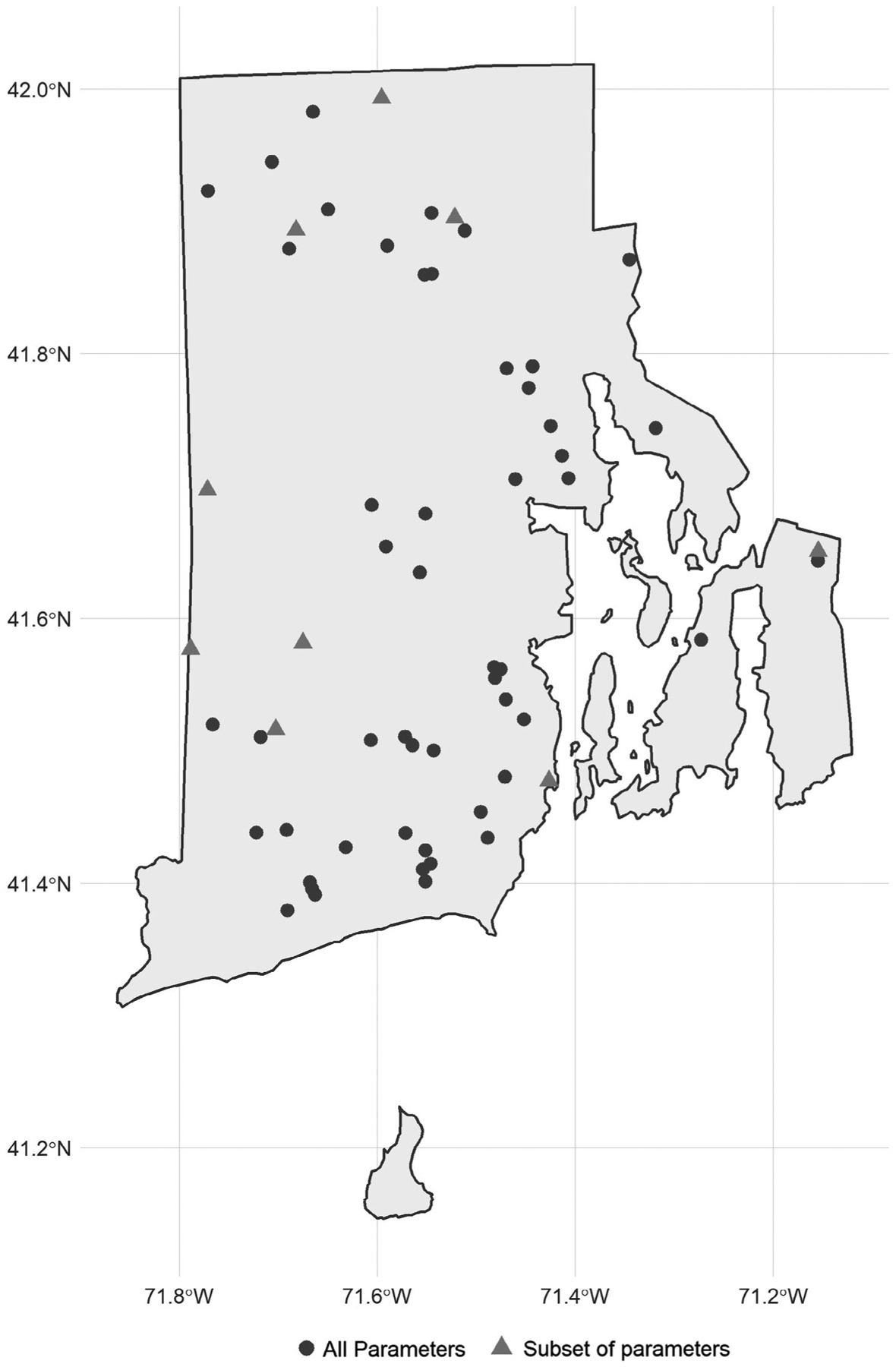
Map of URI Watershed Watch Lake and reservoir sampling sites.

The study area for this analysis includes lakes and reservoirs in the state of Rhode Island where data were collected by the University of Rhode Island’s Watershed Watch program (Fig. 1) which allows for the examination of long-term trends in Rhode Island lakes. The University of Rhode Island’s Watershed Watch (URIWW) is a scientist-led community science program founded in the late 1980s that has built a robust collaboration between URI scientists and a vast network of volunteer monitors. Volunteer monitors are trained and then collected field data (e.g., sonde measurements or Secchi depth) as well as whole water samples during the growing season (e.g., May through October) for later analysis in the laboratory. The entire effort follows rigorous quality control/quality assurance protocols. These types of community science efforts allow for the collection of reliable data that in turn lead to crucial and frequently unexpected insights (Dickinson et al. 2012, Kosmala et al. 2016, Oliver et al. 2017).

The URIWW program began in 1988, monitoring 14 lakes, and has now grown to include over 250 monitoring locations on over 120 waterbodies, including rivers/streams and estuaries, with more than 400 trained volunteers. URIWW now provides more than 90% of Rhode Island’s lake baseline data and is an integral part of the state’s environmental data collection strategy. Data quality assurance and control is treated with paramount importance; volunteers are trained both in the classroom and in the field, regular quality checks occur, and volunteers are provided with all the necessary equipment and supplies, along with scheduled collection dates. For freshwater lakes and reservoirs, weekly Secchi depth and water temperature are recorded, along with bi-weekly chlorophyll *a* and in deep lake (>5 m) dissolved oxygen. Water samples are collected three times per season (May through October) to be analyzed for nutrients and bacteria.

For this analysis, we were interested in trends in lake temperature, TN, TP, TN:TP, and chlorophyll *a*. In particular, we selected URIWW lakes and reservoirs (hereafter, referred to as sites) that matched the following criteria: (1) were sampled between 1993 and 2016; (2) were sampled in May–October; (3) had at least one sampling event per year in May through June, at least one sampling event per year in July through August, and at least one sampling event per year in September through October; (4) had at least one sampling event from 1993 to 2004 and 2005 to 2016; and (5) were sampled at a depth of 2 m or less. As not all sites have data for all selected years, we further filtered the data to select sites that had at least 10 yr of data for a given parameter within the 1993–2016 time frame. The final dataset used in our analysis included 60 lakes and reservoirs. Of these sites, our filtered dataset had 58 sites measured for temperature, 58 sites measured for chlorophyll *a*, 54 sites measured for TN, and 55 sites measured for TP. Of the 60 sampling sites, 51 had data for all five parameters. The N:P ratio was calculated by dividing the mass concentrations of total nitrogen and total phosphorus and then converting to a molar ratio by multiplying by 2.21 (e.g., atomic weight of P 30.974/atomic weight of N 14.007).

Field and analytical methods are detailed on the URIWW website at https://web.uri.edu/watershedwatch/uri-watershed-watch-monitoring-manuals/ and https://web.uri.edu/watershedwatch/uri-watershed-watch-quality-assurance-projectplans-qapps/, respectively. These methods, approved by both the state of Rhode Island and the U.S. Environmental Protection Agency, have remained fairly consistent, although over the nearly 30 yr changes did occur. When new methods were introduced, comparisons between old and new methods were conducted and in all cases no statistically significant differences were found with the new methods. Furthermore, the new methods did at times improve the limits of detection; however, this impacted a very small number (<1%) of measurements in this study. We did run our analyses (see Water quality trend analysis) with all data and with only those data greater than the detection limit. There was no change in the trend analysis, and thus, the results we report are for all data as originally reported in the URIWW dataset. Given these results, we assume the data to be consistent across the reported time period and appropriate for a long-term assessment of trends.

Prior studies have modeled water quality trends across a larger region of the northeastern United States within 17 states including Minnesota, Wisconsin, Iowa, Missouri, Illinois, Indiana, Michigan, Ohio, Pennsylvania, New York, New Jersey, Connecticut, Massachusetts, Rhode Island (which includes the URIWW data), Vermont, New Hampshire, and Maine (Soranno et al. 2015, Oliver et al. 2017). We repeated our analysis (see Water quality trend analysis) with the same dataset used by Oliver et al. (2017), the LAGOS-NE dataset (Soranno et al. 2015, 2017, 2019, Stachelek and Oliver 2020). Temperature data were not available; thus, we examined trends, using our analytical methods, for TN, TP, TN:TP, and chlorophyll *a* from the LAGOS-NE dataset. We used the same selection criteria on the LAGOS-NE dataset as was applied to the URIWW data. This ensured that both datasets represented the same seasonal time frame. Exact measurement depth for the LAGOS-NE data was not available; however, the water quality data are reported to be either surface or epilimnion samples (Soranno et al. 2017). At the time of our analysis, the LAGOS-NE data do not extend beyond 2013 thus sites needed to have a sampling event in 1993–2002 and in 2003–2013 to be included.

We use data, also from LAGOS-NE, on landscape composition, maximum lake depth, and lake area (Soranno et al. 2017). We use the 500-m buffer landscape composition for three classes from the 2011 National Land Cover Dataset: Agriculture, Forest, and Developed. The maximum lake depth is from various sources, and lake area is calculated from the National Hydrography Dataset waterbody polygons. Additional details are available in Soranno et al. (2017).

Lastly, data and code for the entire analysis are available from https://github.com/USEPA/ri_wq_trends/ and are archived at https://doi.org/10.5281/zenodo.4306765 (Hollister et al. 2020). All analyses were conducted with R version 4.0.4, and details on R package versions and operating system used for this analysis are included in a file, sessioninfo.txt at https://github.com/USEPA/ri_wq_trends/blob/master/sessioninfo.txt (Grolemund and Wickham 2011, McLeod 2011, Alathea 2015, Xie 2015, 2021, Pebesma et al. 2016, Wickham 2016, 2019, 2021, Daróczi and Tsegelskyi 2018, Mullen 2018, Mullen and Bratt 2018, Pebesma 2018, Wickham and Bryan 2019, Bocinsky 2020, Müller 2020, Rudis 2020, Wickham and Hester 2020, Wilke 2020, Gohel 2021, Hollister et al. 2021, Ooms 2021, R Core Team 2021, Robinson et al. 2021, Walker and Herman 2021, Wickham et al. 2021). Values included in each of the figures have also been separately saved as a comma-separated value file, yearly_average_anomaly.csv, and may be retrieved via https://github.com/USEPA/ri_wq_trends/blob/master/data/yearly_average_anomaly.csv.

### Water quality trend analysis

There are many different methods for analyzing time series data for trends. Environmental data are notoriously noisy and one of the difficulties that is encountered with multiple sampling sites is how to identify a trend while there is variation within a sampling location as well as variation introduced by differing start years for sampling among the many sites. For instance, if long-term data on water quality were collected more frequently in early years from more pristine waterbodies, then a simple comparison of raw values over time might show a decrease in water quality, which could be misleading if later sampling occurred on both pristine and more degraded water bodies. Thus, it is necessary to account for this type of within-site and amongsite variation. To do so, we used methods similar to those used to analyze long-term temperature trends using temperature anomalies (e.g., Jones and Hulme 1996). The general approach we used calculates site-specific deviations from a long-term median over a pre-determined reference period. This is slightly different than the typical use with temperature anomalies as those usually use the mean instead of the median. Many of the variables we looked at are non-normal and often have outliers (e.g., algal blooms). In this case, the median is preferred. Using the median with site-specific deviations allows all sites to be shifted to a common baseline and the deviations, or anomalies, indicate change over the specified reference period. We refer to this method as “site-specific anomalies.”

#### Summarizing site-specific anomalies.—

**Fig. 2.**
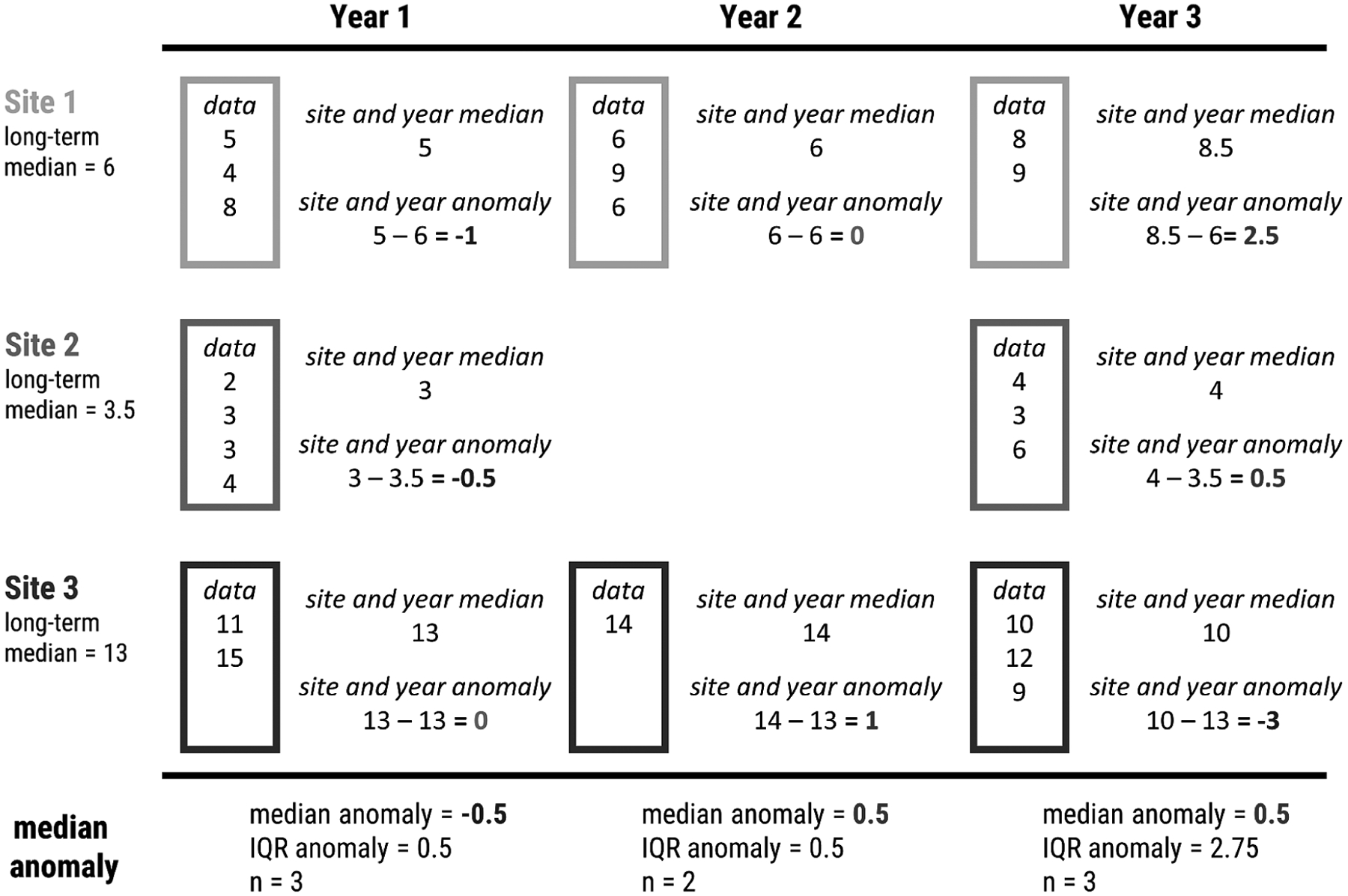
Example calculation of the site-specific anomalies and yearly median anomalies.

Methods for calculating the site-specific anomalies and the yearly medians are as follows and are presented graphically in Fig. 2. Additionally, an example R script, schematic_anomaly.R and example dataset, schematic.csv to recreate and demonstrate the calculations in Fig. 2 is available from https://github.com/usepa/ri_wq_trends/blob/master/R/schematic_anomaly.R and is archived at https://doi.org/10.5281/zenodo.4306765 (Hollister et al. 2020).

The general steps, outlined in Fig. 2 and listed below, are repeated for each of the water quality parameters.

1. For each site, calculate the annual medians, producing a single median value for each site and year. This step prevents bias from pseudoreplication of multiple measurements of the same site in a given year (Hurlbert 1984). The per site medians across years are assumed to be independent.
2. Calculate the long-term reference median for each site. This results in a single site-specific long-term median.
3. Calculate the anomaly for each annual median at each site by subtracting the sites reference long-term medians from the sites yearly median.
4. Summarize by calculating the median anomaly per year for the entire group of sites. The resultant values are analyzed for a trend over time.

**Fig. 3.**
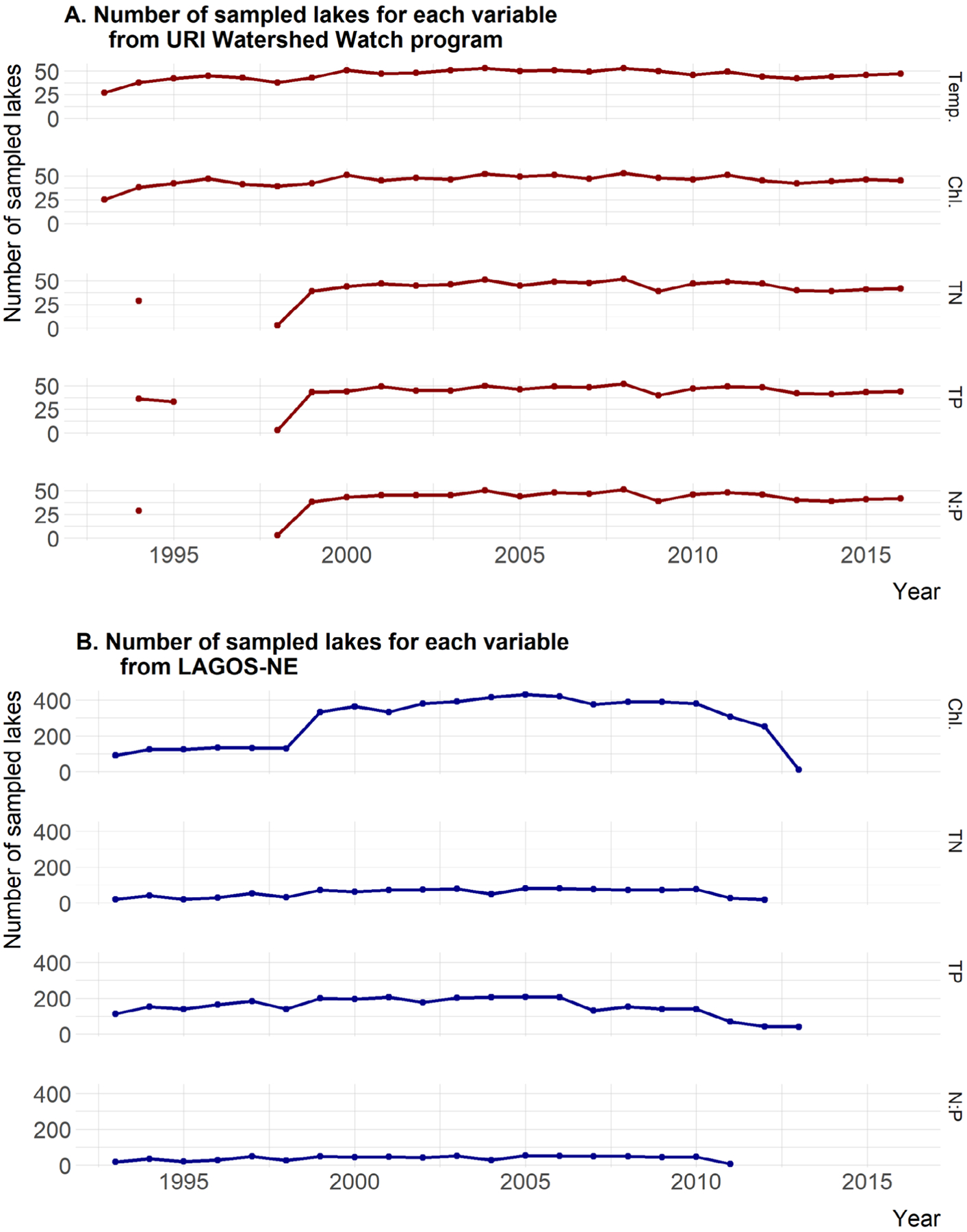
Number of sites available for trend analysis by parameter and year for both URIWW and LAGOS-NE.

After filtering and summarizing the data, some years may not have sufficient number of sites to be included. We chose to include years in the analysis if they had at least three sites, but years with small numbers of sites are rare and only occurred with the nutrient data very early in the time frame of our analysis for the URIWW data and late in the time frame for the LAGOS-NE data (Fig. 3).

#### Linear regression on annual median anomalies.—

Testing for a regression slope being different than zero can be used to test for monotonic trends in water quality data (Helsel and Hirsch 2002). We used these standard procedures to look for positive or negative trends in lake temperature, chlorophyll *a*, TN, TP, and TN:TP. For each parameter, we fit a regression line to the anomalies as a function of year and tested the null hypothesis that no trend existed (e.g., β_1_ = 0). The slope of this line provides information on the average yearly change of that parameter over the time period studied.

#### Assessing regressions for trends.—

Traditionally, trends would be determined by assessing significance but recent guidelines suggest not using arbitrary *P* value cut-offs to assesses significance (Wasserstein et al. 2016). Our interpretation of the trends attempts to follow this advice, and we assess trends with multiple lines of evidence. For this assessment, we evaluated trends based on the following criteria: (1) *P* values are used to determine general levels of statistical support, (2) relative frequency of high and low years in the beginning and end of the time frame are used to identify an increasing or decreasing pattern, and (3) the magnitude of the slope is used to infer an ecologically relevant change. Using all of this information, we determine that a trend exists if it meets at least 2 of the 3 criteria, a weak trend exists if it meets one of the criteria, and no trend exists if it meets none of the criteria.

We recognize that ecologically relevant change is system-dependent and no single value will be universally appropriate. We are using several values for this particular assessment, but other values could also be justified. There are various ways to identify important temperature changes in lakes and reservoirs. A policy-based approach could examine agreements that attempt to limit temperature increases, such as the Paris Agreement, which aims to limit increases over the next century to 2°C, which would be 0.02°C per year or, the state of Rhode Island criteria for temperature increase is 2.2°C or 0.022°C per year over a century (RIDEM 2010, UN 2015). Alternatively, we could look at more ecologically focused temperature changes. For instance, Winder and Schindler (2004) saw disrupted trophic linkages between phytoplankton and zooplankton with 1.39°C over a 40-yr data set, or 0.035°C per year. For this study, we will use the 0.02°C per year as it would provide protections against other ecologically relevant changes (e.g., 0.035°C per year for trophic linkages), meet local criteria, and would also be indicative of meeting an important policy goal.

For changes in chlorophyll, total phosphorus, and total nitrogen, we define an ecologically meaningful trend as one that would result in any oligotrophic lake changing to a mesotrophic state over the course of a century. We use the values defined by the trophic state limits in Nürnberg (1996). For chlorophyll, the oligotrophic–mesotrophic limit is 3.5 μg/L and over a century that is 0.035 μg/L per year; for total nitrogen, the limit is 350 μg/L or 3.5 μg/L per year, and the total phosphorus limit is 10 μg/L or 0.1 μg/L per year. A slope at or above these values would result in a oligotrophic–mesotrophic transition for all oligotrophic lakes, even lakes with the impossible situation of chlorophyll, total nitrogen, or total phosphorus concentrations of zero.

An ecologically meaningful trend for TN:TP could be one that would suggest a switch from nitrogen limitation to phosphorus limitation over a century. To identify this, we use information from experimental manipulations of nitrogen in lakes that identify those lakes as being nitrogen limited (Downing and McCauley 1992). In Downing and McCauley (1992), they identified 34 studies and reported whether or not the study found the lake to be nitrogen limited and the ambient total nitrogen and total phosphorus concentration in μg/L. With this information, we calculated a median molar TN:TP ratio for nitrogen limited lakes and for lakes that did not show nitrogen limitation. The difference between these two values would suggest a possible shift in the limiting nutrients. The median molar TN:TP for nitrogen limited lakes was 35 and for lakes not limited by nitrogen was 61. The difference between these is 26 and over a century a change of 0.26 per year would be indicative of an ecologically meaningful change. As we have defined it, slope magnitudes in excess of these per year values (temperature: 0.02, chlorophyll: 0.035, total nitrogen: 3.5, total phosphorus: 0.1, TN:TP: 0.26) will be considered to have an ecologically meaningful trend.

#### Comparison of Rhode Island to the region.—

Oliver et al. (2017) used hierarchical linear models and showed relatively stable water quality in the lakes of the northeastern United States. While the University of Rhode Island’s Watershed Watch data were included in this regional study, we hypothesized that, in the case of Rhode Island, regional trends were masking sub-regional trends. Therefore, we decided to reanalyze the LAGOS-NE data to compare the trends at the regional scale to the trends at the Rhode Island state scale using the site-specific anomaly and trend analysis approach outlined above.

## Results

Lakes and reservoirs in Rhode Island tended to be smaller and shallower, on average, than lakes included in the complete LAGOS-NE region (Table 1). Also, Rhode Island lakes tended to occur in landscapes (i.e., a 500-m buffer) with a lower average percent agriculture, more developed land, and comparable forested area than lakes in the LAGOS-NE region (Table 1).

During the period of 1993–2016, Rhode Island lakes and reservoirs in our dataset of surface and epilimnion measurements had a median lake temperature of 23°C, median TN of 460 μg/L, median TP of 15 μg/L, median TN:TP ratio of 68.1 mol/L, and median chlorophyll *a* of 3.5 μg/L (Table 2).

For lakes and reservoirs in the larger region represented by the LAGOS-NE States, median TN was 560 μg/L, median TP was 16 μg/L, median TN:TP ratio was 61.95 mol/L, and median chlorophyll *a* was 6.5 μg/L (Table 3).

On average, the Rhode Island lakes and reservoirs show lower concentrations of nutrients with more nitrogen relative to phosphorus than in the larger LAGOS-NE region (Tables 2, 3). Chlorophyll concentrations also show lower average concentrations in Rhode Island (Tables 2, 3). Furthermore, the distribution of chlorophyll-based trophic state also shows a larger percentage of oligotrophic and mesotrophic lakes in Rhode Island and more eutrophic and hypereutrophic lakes in the larger LAGOS-NE region (Table 4; Nürnberg 1996). These results follow what would be expected given that, on average, LAGOS-NE lakes are more dominated by agricultural lands than are Rhode Island lakes (Table 1).

### State-wide trends in water quality

**Fig. 4.**
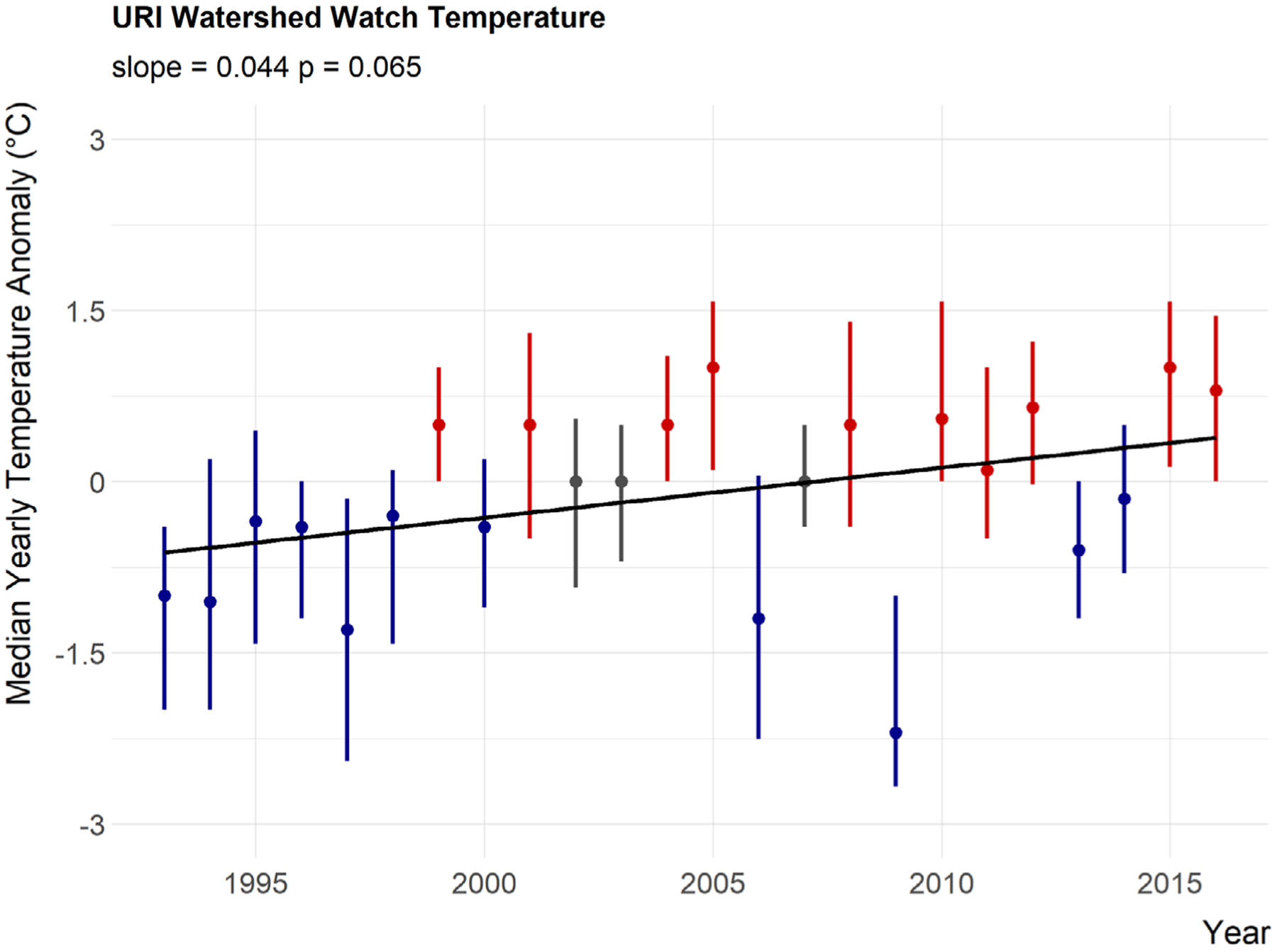
Twenty-year trend for median lake temperature anomaly in Rhode Island. Points are medians of site-specific anomalies and ranges are the 25th and 75th percentiles. Blue indicates yearly site-specific anomalies that were below the site-specific long-term medians. Red indicates yearly site-specific anomalies that were above the site-specific long-term medians. Black line is the linear fit for year and median anomaly.

**Fig. 5.**
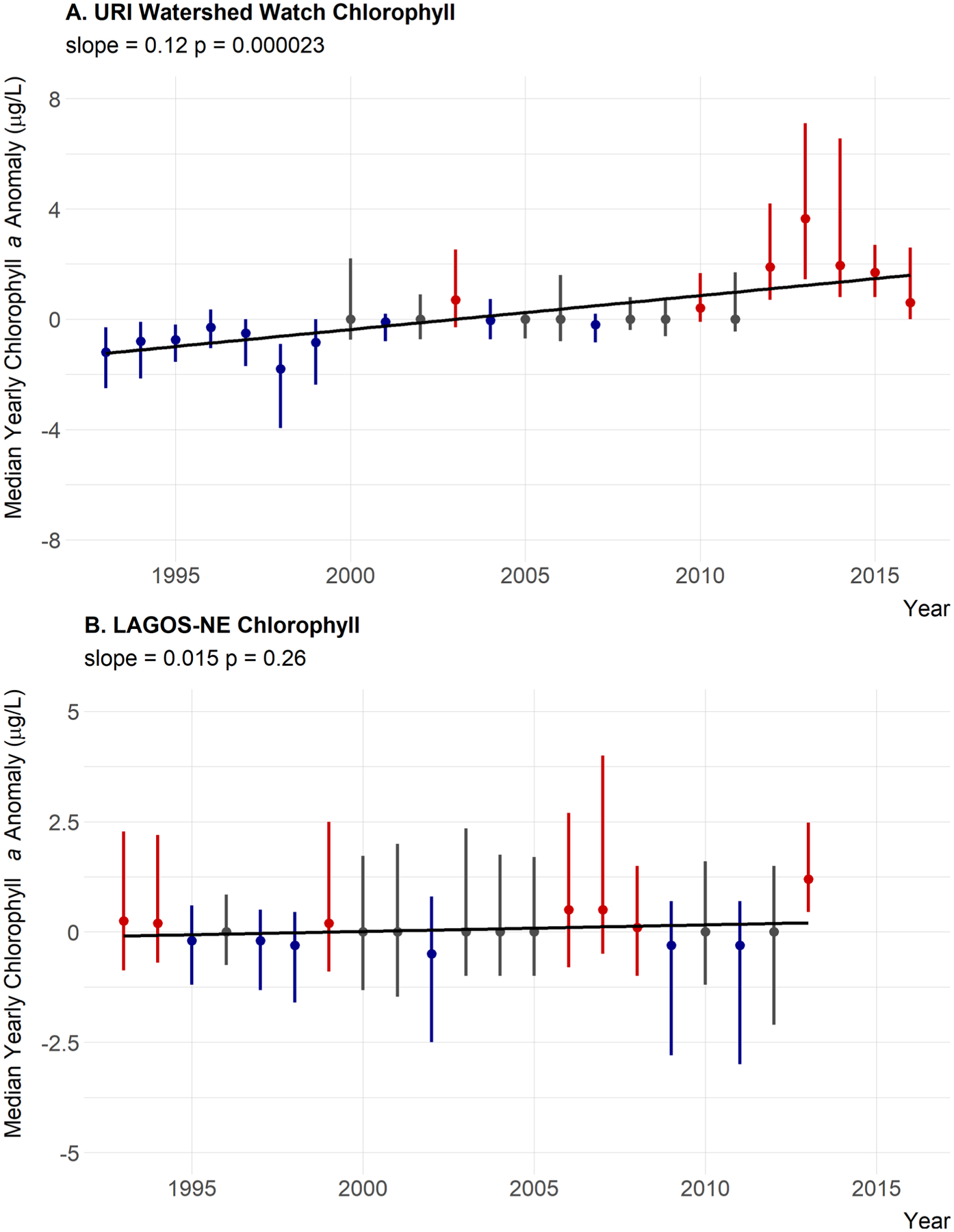
Twenty-year trend for median chlorophyll anomaly. (A) URI Watershed Watch yearly chlorophyll anomalies. (B) LAGOS-NE yearly chlorophyll anomalies. Points are medians of site-specific anomalies and ranges are the 25th and 75th percentiles. Blue indicates yearly site-specific anomalies that were below the site-specific long-term medians. Red indicates yearly site-specific anomalies that were above the site-specific long-term medians. Black line is the linear fit for year and median anomaly.

Median annual temperature anomalies in lakes and reservoirs appear to be increasing as the slope is greater than the threshold of 0.02 we identified, the *P* value suggests some statistical support for a positive trend (slope = 0.044, *P* = 0.065), and the majority of years with median temperature greater than the long-term median are occurring in the second half of the time period (Fig. 4; Table 5). Chlorophyll *a* is also showing an increasing trend over time (slope = 0.12, *P* = 0.000023). The slope of 0.12 μg/L is greater than our threshold of 0.035 μg/L and the above-average years have mostly occurred in the most recent years (Fig. 5A; Table 5).

**Fig. 6.**
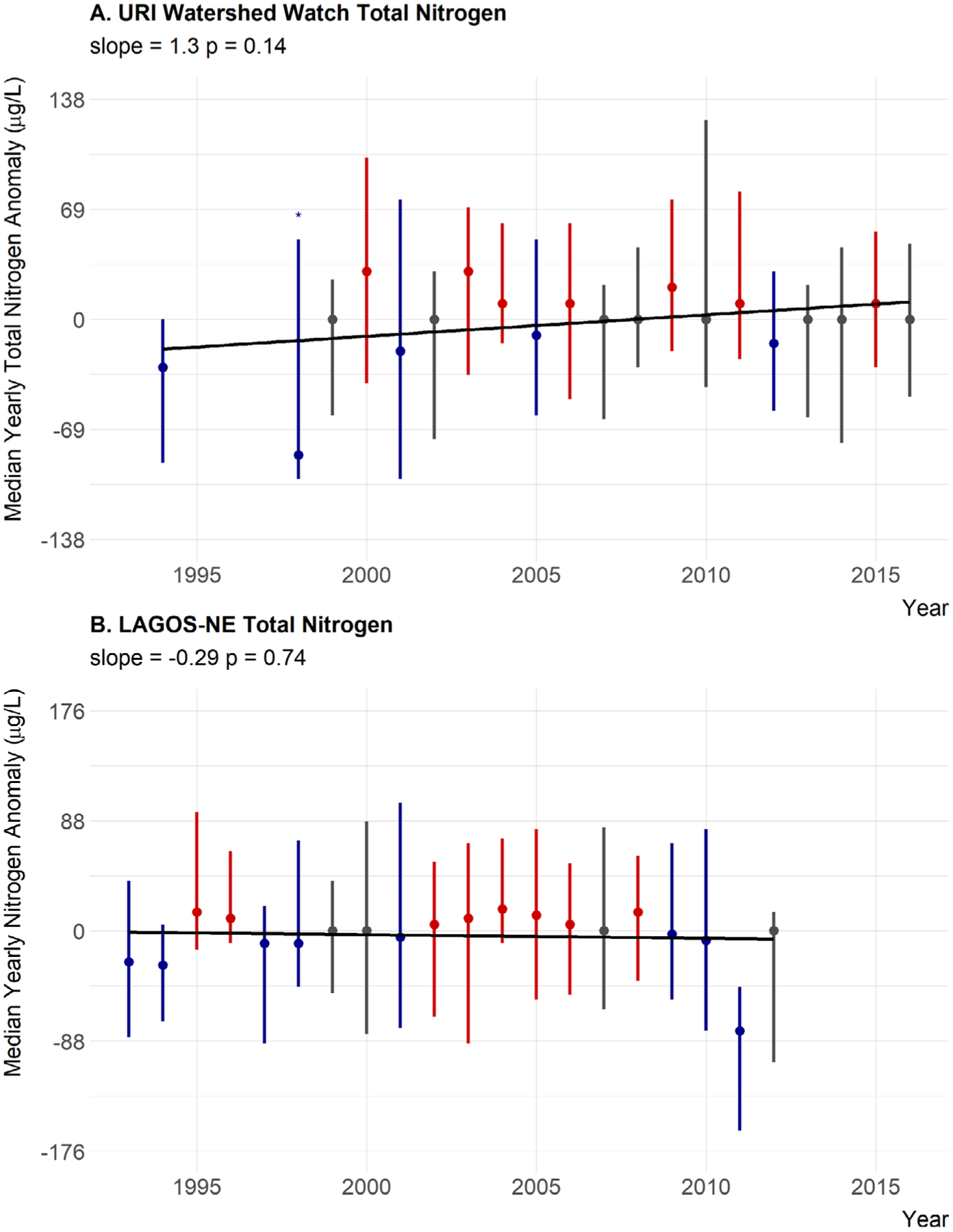
Twenty-year trend for median TN anomaly. (A) URI Watershed Watch yearly TN anomalies. (B) LAGOS-NE yearly TN anomalies. Points are medians of site-specific anomalies and ranges are the 25th and 75th percentiles. Blue indicates yearly site-specific anomalies that were below the site-specific long-term medians. Red indicates yearly site-specific anomalies that were above the site-specific long-term medians. Gray indicates yearly site-specific anomalies that were equal to the long-term medians. Missing years had insufficient data, an asterisk indicates years with only three sites, and error bars are the range of the data. Black line is the linear fit for year and median anomaly.

**Fig. 7.**
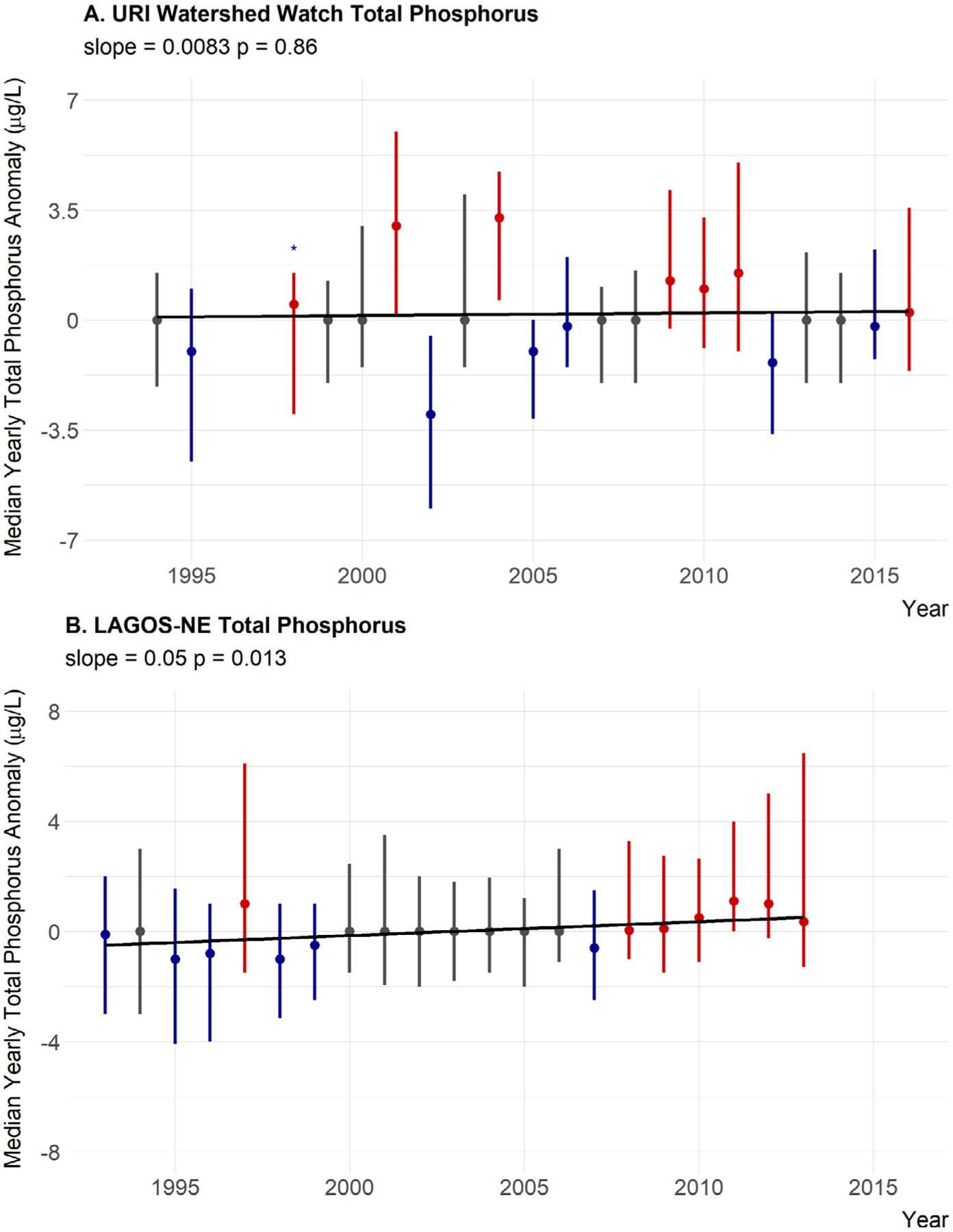
Twenty-year trend for median TP anomaly. (A) URI Watershed Watch yearly TP anomalies. (B) LAGOS-NE yearly TP anomalies. Points are medians of site-specific anomalies and ranges are the 25th and 75th percentiles. Blue indicates yearly site-specific anomalies that were below the site-specific long-term medians. Red indicates yearly site-specific anomalies that were above the site-specific long-term medians. Gray indicates yearly site-specific anomalies that were equal to the long-term medians. Missing years had insufficient data, an asterisk indicates years with only three sites, and error bars are the range of the data. Black line is the linear fit for year and median anomaly.

**Fig. 8.**
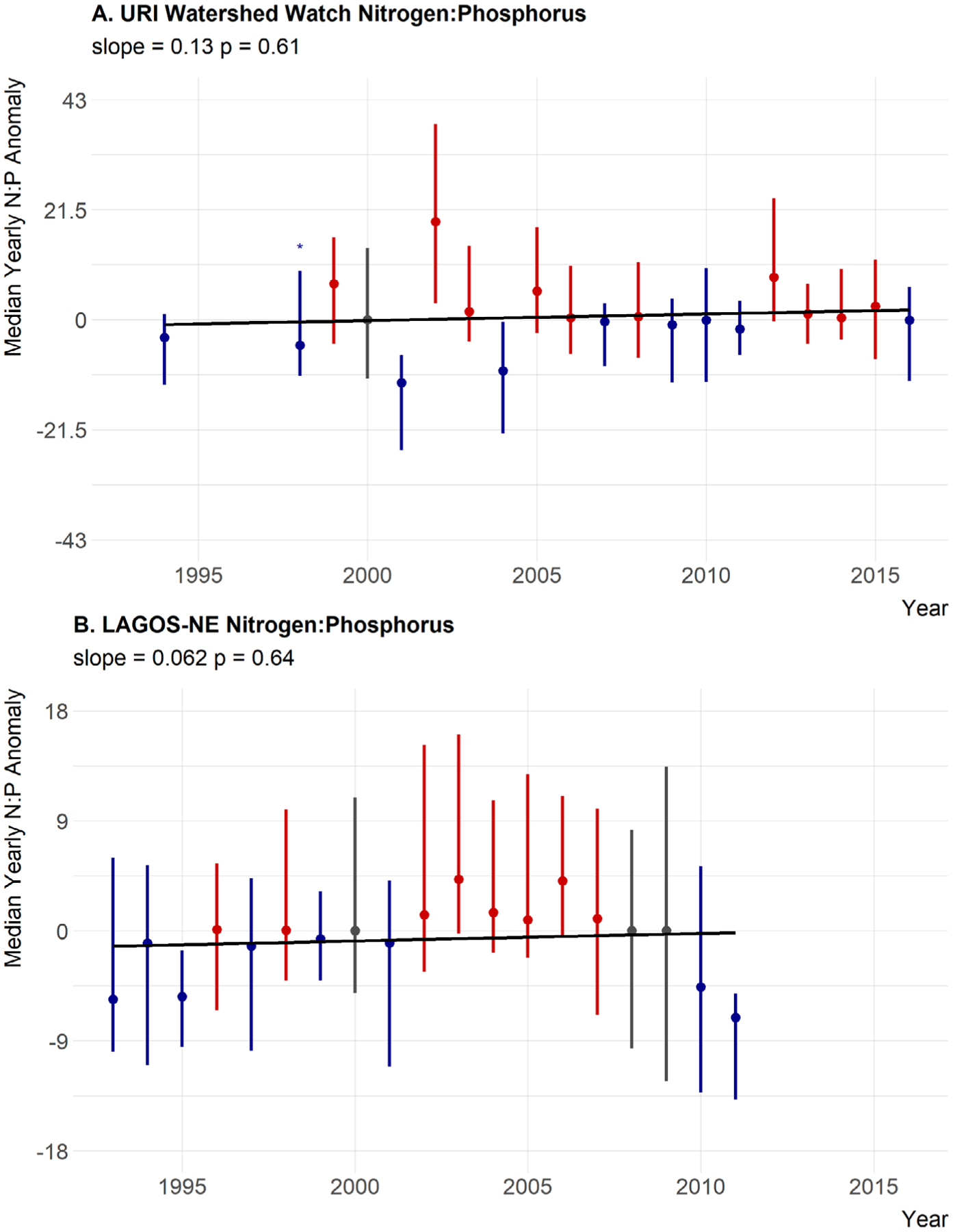
Twenty-year trend for median TN:TP anomaly. (A) URI Watershed Watch yearly TN:TP anomalies. (B) LAGOS-NE yearly TN:TP anomalies. Points are medians of site-specific anomalies and ranges are the 25th and 75th percentiles. Blue indicates yearly site-specific anomalies that were below the site-specific long-term medians. Red indicates yearly site-specific anomalies that were above the site-specific long-term medians. Gray indicates yearly site-specific anomalies that were equal to the long-term medians. Missing years had insufficient data, an asterisk indicates years with only three sites, and error bars are the range of the data. Black line is the linear fit for year and median anomaly.

Median annual trends for nutrients were not as clear. For total nitrogen, the slope of the line is positive with some, albeit weak, statistical support (slope = 1.3, *P* = 0.14) but the years greater than the median are distributed evenly throughout time. Also, a slope of 1.3 μg/L per year is lower than our threshold value of 3.5 μg/L suggesting little support for a meaningful ecological change over time (Fig. 6A). Also, 1998 only had three sites with available total nitrogen data and may not be representative. This year also recorded the lowest median value. Thus, 1998 may be skewing these results. When this year is removed, the slope of the line is 0.39 and the *P* value is 0.53. Thus, we interpret this as no trend in total nitrogen (Table 5). Total phosphorus shows essentially no trend over time in the yearly anomalies with little statistical support (slope = 0.0083, *P* = 0.86), and years that are over the median do not show any pattern (Fig. 7A; Table 5). Also a change of 0.0083 μg/L per year is much less than our defined threshold of 0.1 μg/L. The TN:TP ratio has a small slope, very little statistical support, no pattern in the above and below years, and a slope magnitude less than 0.26 (slope = 0.13, *P* = 0.61) suggesting little evidence for a change in the concentrations of TN relative to the concentrations of TP (Fig. 8A; Table 5).

### Regional trends in water quality

In general, there was little evidence to suggest broad regional changes in chlorophyll *a* as it showed a very weak positive trend, slight statistical support, and above-average years spread evenly throughout the time period (slope = 0.015, *P* = 0.26; Fig. 5B; Table 5). Total nitrogen showed a slight decreasing trend with weak statistical support and slope less than the 3.5 μg/L that would result in a trophic state change over a century; thus, there is little support for a meaningful trend in TN at the regional scale (slope = −0.29, *P* = 0.74; Fig. 6B; Table 5). Furthermore, the last two years for which LAGOS-NE had data on total nitrogen were from a relatively smaller number of sites (Fig. 3) and those values may not be representative. Total phosphorus showed a very small increasing trend, and there is statistical support for that trend. The slope however suggests little support for an ecological meaningful change in total phosphorus (slope = 0.05, *P* = 0.013; Fig. 7B; Table 5). Lastly, the TN:TP ratio showed little change (slope = 0.062, *P* = 0.64; Fig. 8B, Table 5) as none of our criteria for a trend were met. Although, there appears to be a non-linear trend that suggests increasing TN:TP early in the time period and decreasing TN:TP later, we feel we cannot effectively evaluate this though as total nitrogen measurements were rare in 2011 (Fig. 3) and sites that had both nitrogen and phosphorus were even rarer. This low median anomaly should be interpreted with this caveat in mind. Taken together we feel our results largely match the findings of Oliver et al. (2017) that there is stasis in lake nutrients and chlorophyll within the LAGOS-NE region.

## Discussion and Conclusions

Our sub-regional analysis indicates that increases in primary production, as measured by chlorophyll *a*, occurred over the ~25 yr of our study period. Over the same period, we also demonstrate long-term warming of Rhode Island lakes and reservoirs. Chlorophyll has increased, on average, 0.12 μg/L per year over the 23 yr of our analysis, while temperature has increased 0.044°C per year over the same period. Trends in nutrients suggested little change. Total nitrogen, total phosphorus, and the nitrogen:phosphorus ratio all showed no evidence of trends. While our analysis is not capable of detecting causation, both chlorophyll *a* and temperature is increasing with less obvious trends in nutrients is interesting and warrants further exploration to see if increasing chlorophyll *a* can be described by temperature. Also, geographic extent does indeed matter when trying to identify long-term water quality trends. Similar to the results of Oliver et al. (2017), our analysis shows little increasing trend in chlorophyll *a* at the regional scale (e.g., northeastern and midwestern United States). However, at the more localized scale of the state of Rhode Island, there is a clear increasing trend in chlorophyll *a*.

### Trends

As previously mentioned, both temperature and chlorophyll *a* show increasing trends from 1993 to 2016 in Rhode Island lakes and reservoirs (Table 5). However, 2006 and 2009 stand out as not following this trend for temperature. Average May–October air temperature for 2009 was third lowest on record for the 1993–2016 time period and also showed the lowest maximum temperatures (NOAA NCEI 2020). On the other hand, 2006 was not unusually cool (11th lowest out of 24 yr) but it was the wettest year on record for this period (NOAA NCEI 2020). These unusual weather patterns may help explain why 2006 and 2009 did not follow the trend.

Trends in total nutrients and the TN:TP ratio are less clear. While TN showed a weak positive trend, data for the early years (1994–1998) were sparse. There is no ecological or statistical trend in the years with consistently available data. The general picture in Rhode Island appears to be no increase in nitrogen, no trend in phosphorus, and little to no change in the TN:TP ratio (Table 5). We interpret these results as relative stability in nutrients in Rhode Island lakes and reservoirs.

Stable nutrient regimes may be partly explained by efforts to curb nutrient loadings, for example, through voluntary and state-wide mandatory bans on phosphates in laundry detergent which were implemented in Rhode Island in 1995 (Rhode Island State Legislature 1995, Litke 1999). However, these nutrient reductions may not result in nutrient limitation and when faced with other changes (e.g., temperature increases) increasing chlorophyll *a* may still occur. Additionally, our analysis points to the fact that chlorophyll *a* and algal biomass is driven by processes operating at different scales. For instance, nutrient management is largely a local to watershed scale effort, but may also be regional as atmospheric nitrogen deposition can be a significant source of nitrogen (Boyer et al. 2002). Similarly, warming lakes are driven by broader climate patterns, yet waterbody-specific factors such as the percent of impervious surface in a catchment and lake morphology can also impact temperature (Nelson and Palmer 2007). In short, differences in regional- and state-level trends are driven by complex and multi-scale processes.

In addition to the sub-regional annualized trends of the five variables, we address with this study, other trends may also be of interest. For example, a lengthening of the growing season could increase the number of days with conditions that favor the growth of harmful algal blooms as cyanobacteria grow better at temperatures greater than 25°C (Reynolds 2006, Jöhnk et al. 2008, Paerl and Huisman 2008). Many national and regional studies have also documented longer growing seasons (Cooter and Leduc 1995, Kunkel et al. 2004, e.g., Vega et al. 2019). Evidence in Rhode Island also points to possible lengthening of the growing season as both May and October, the beginning and end of the sampling dates in our dataset, show increasing temperature trends over time (NOAA NCEI 2020). Furthermore, preliminary analysis of the URIWW data supports the idea that growing seasons may be getting longer in Rhode Island. Early in our study period (1993–1995), the average first day of lake temperatures exceeding 25°C was June 11; at the end of our study period (2014–2016), these warmer temperatures were seen, on average, on June 1. A site-specific anomaly analysis of growing season length could shed further light on potential changes to growing seasons in Rhode Island lakes.

Trends, and their ecological importance, could also vary depending on trophic state of a given waterbody. We identified a minimum ecologically important change of 0.035 μg/L per year of chlorophyll. This magnitude of change would result in a shift for any oligotrophic lake to a mesotrophic state, whereas for any mesotrophic lake to become eutrophic over a century would require a change of 0.055 μg/L per year. Examining these trends by trophic state provides the ability to look for ecologically important changes across the range of trophic state and not rely only on a single rate for all lakes. Furthermore, Stoddard et al. (2016) report an 18.2% reduction in the number of oligotrophic lakes in the United States from 2007 to 2012. A site-specific anomaly analysis by trophic state would identify trends, such as those seen in Stoddard et al. (2016), that might be occurring only on oligotrophic lakes. A full analysis and interpretation of trends by trophic state is beyond the scope of this study. However, we conducted a preliminary analysis of chlorophyll trends in Rhode Island for oligotrophic and hypereutrophic lakes which showed increasing chlorophyll trends for both trophic states but the yearly increase was much greater in the hypereutrophic lakes. This suggests that a full analysis of how lakes in different trophic states are changing over time is warranted.

### Broader implications

There are several broader implications from the results of our analysis and of examining long-term water quality trends in general. First, as more long-term datasets become available, it is important for managers, stakeholders, and researchers to work together to better understand long-term water quality trends at multiple spatial scales. Specifically for this study, the results provide feedback to long-time volunteer monitors about the trends in long-term, volunteer-collected data. This type of feedback is important in maintaining involvement as it has been shown in other areas that contributing to science and management is one of the expectations for some community science volunteers and can help maintain longer term involvement (Gouveia et al. 2004, Bonney et al. 2009, Ganzevoort et al. 2017). If long-term involvement was not common, then consistent long-term data may be difficult to obtain and understanding of long-term trends would be challenging.

Second, with information on long-term trends, it is possible to adapt management approaches to address areas of concern. Our results show increasing chlorophyll *a* and increasing temperature even though the general long-term nutrient trends have been less clear. While the analysis described here can not be used to infer causation, it points to areas that may need to be addressed. For instance, warming waters are linked to increases in harmful algal blooms (Paerl and Huisman 2008, 2009, Paerl and Paul 2012). An increase in blooms could be assumed via increasing chlorophyll *a* levels as chlorophyll *a* has been linked to probability of the presence of bloom indicators such as microcystin (Hollister and Kreakie 2016). Our results are consistent then with the prior research that temperature changes could be driving increased chlorophyll.

Our analysis has shown warming lakes in Rhode Island, but broad-scale warming of lakes has also been documented and shown to be a result of both climatic and local drivers and can vary greatly within regions (e.g., O’Reilly et al. 2015). Given that the drivers of warming are at both broad and local scales, managing warming lake temperatures will be a difficult task. Counteracting the impacts of continued warming on increased chlorophyll in lakes will, at a minimum, requires additional interventions. Reducing nutrient loads below current levels is one such intervention and could be achieved via source controls, enhanced entrainment of surface waters, treatment through green infrastructure, or in-lake approaches such as restoration of freshwater mussels (Kellogg et al. 2010, Pennino et al. 2016, Kreeger et al. 2018, Yang and Lusk 2018, Reisinger et al. 2019). In any event, controlling future eutrophication and protecting against harmful aglal blooms will continue to be a challenge against the backdrop of warming lakes.

### Data analysis approach

The analysis approach we used here, site-specific anomalies, is not a novel method and does have a long history in the analysis of trends in climate (Jones and Hulme 1996, Jones et al. 1999, Hansen et al. 2006, 2010). However, using it to examine water quality trends is a little-used application of the technique, as we only found a single study using anomalies in lakes and that study’s focus was only on lake temperature (O’Reilly et al. 2015). There is little evidence of using anomalies more broadly with water quality trends. Thus, we built on these methods and adapted them for use with long-term water quality trends. While other methods are valid and robust (e.g., Oliver et al. 2017), we chose median site-specific anomalies as they can provide readily interpretable results, especially for communicating to general audiences. For instance, reporting the changes in anomalies allows us to look at changes in the original units. With our analysis, the slope of the regression line for temperature suggests an average yearly increase of 0.044°C and the slope of the regression line for chlorophyll *a* shows an average yearly increase of 0.12 μg/L.

**Fig. 9.**
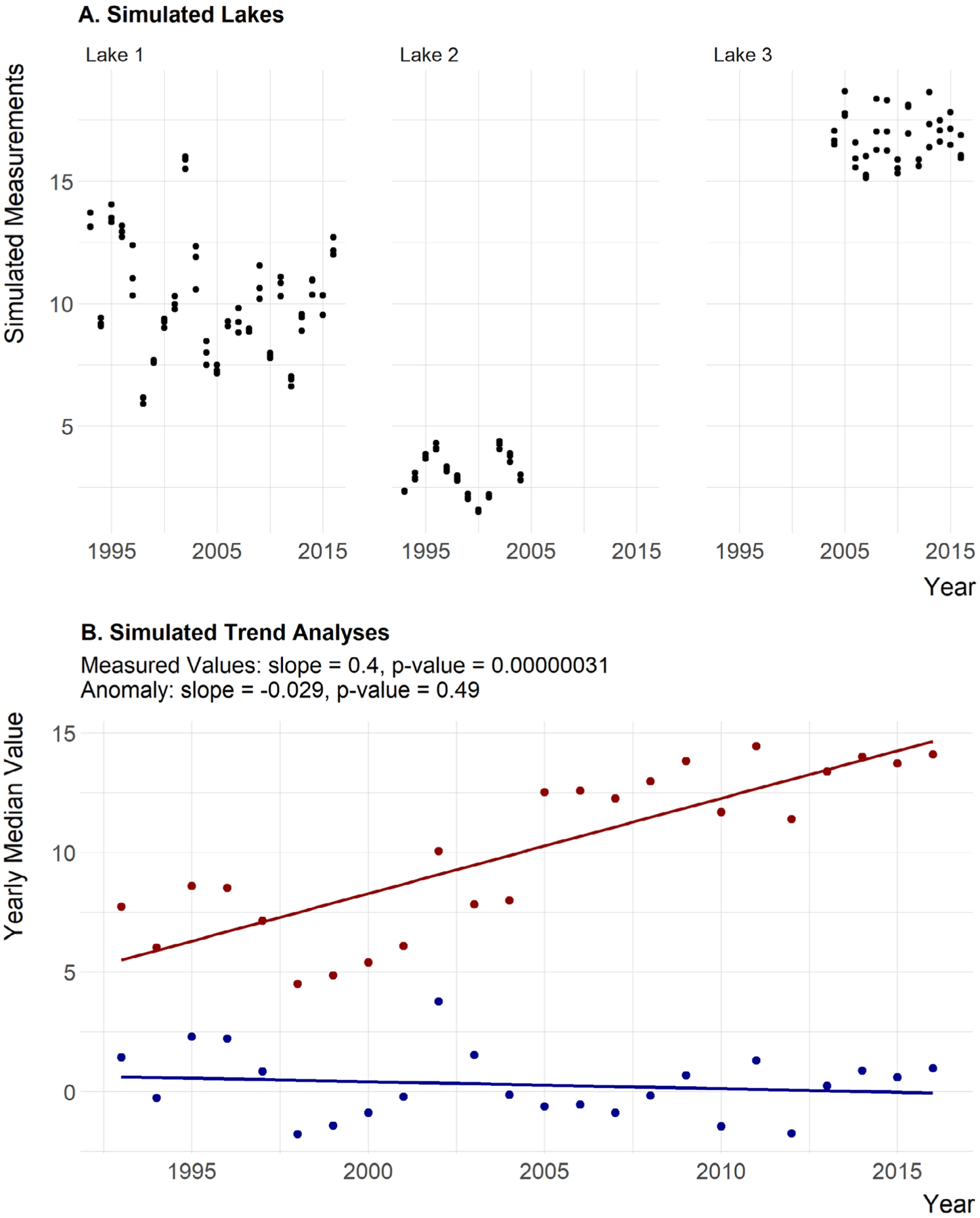
(A) Simulated, random data showing example of three hypothetical lakes; one lake was monitored throughout the record, one lake with low reported values monitored early in the record, and another lake with high reported values monitored late in the record. (B) Analysis of simulated, random data with one lake monitored throughout the record, one lake with low reported values monitored early in the record, and another lake with high reported values monitored late in the record. Yearly average of the actual values is shown in red, and yearly average of the site-specific anomalies is shown in blue.

The site-specific anomalies are also robust to variations in sampling effort and in the timing of inclusion of given sampling locations. For instance, if a site included only early in a time period had low values and a site included late in the time period had high values, then analyzing the measured values over this time period would show an increase whether or not one existed. Using the site-specific anomalies rescales the values and allows the real trend to be seen. We illustrate this with simulated, random data (e.g., no site-specific trend; Fig. 9A) and apply the site-specific analysis to those simulated data (Fig. 9B). The average of the yearly measured values shows a trend (slope = 0.4 and *P* = 0.0000003), whereas the site-specific anomalies correctly show no trend as each site did not change over time (slope = −0.029 and *P* = 0.49; Fig. 9).

Site-specific anomalies do have broad utility for the analysis of water quality trends; however, there are a few caveats the must be considered. Our use of site-specific anomalies as outlined in this paper requires aggregating over years. Any time period could be used for aggregating, but this is a decision that will need to be carefully addressed when conducting this type of analysis. Site-specific anomalies also require fairly large amounts of data. The required data will need to be consistently collected over a long period of time. Ideally, the data would cover a reference period (e.g., for our analysis that was 24 yr) although other data could possibly be used to estimate the long-term medians. In short, site-specific anomalies do require relatively large datasets and decisions on appropriate levels of aggregation, as with any analysis using aggregation, must be made carefully.

Lastly, this analysis is only possible because of the availability of sound, long-term data on water quality in Rhode Island. Without the URIWW data and the commitment and participation of more than 2500 volunteers over the years, our analyses would have been impossible. Going forward, it is important to appreciate the role that volunteer monitoring and community science programs can play in capturing and better understanding long-term environmental trends.

## Figures and Tables

**Table 1.** Average landscape context and lake morphometry summary statistics for lakes in URIWW and LAGOS-NE.

Source	Agriculture (%)	Developed (%)	Forest (%)	Lake area (ha)	Max. depth (m)
URIWW	5.6	27.5	40.7	15.1	5.1
LAGOS-NE	19.3	11.3	41.2	27.9	9.6

**Table 2.** Summary statistics for URI Watershed Watch data from 1993 to 2016.

Parameter	Units	25th percentile	Mean	Median	75th percentile	Max	SD
Temperature	°C	21.9	22.80	23.0	24.0	27.0	1.9
Total nitrogen	μg/L	370.0	580.00	460.0	660.0	4415.0	375.0
Total phosphorus	μg/L	10.0	22.00	15.0	22.0	373.0	28.0
N:P	mol/L	51.0	80.21	68.1	88.4	1326.0	69.5
Chlorophyll	μg/L	2.0	7.50	3.5	7.6	134.5	11.7

**Table 3.** Summary statistics for LAGOS-NE data from 1993 to 2016.

Parameter	Units	25th percentile	Mean	Median	75th percentile	Max	SD
Total nitrogen	μg/L	370.00	725.00	560.00	890.0	10,100.0	680.00
Total phosphorus	μg/L	11.00	29.00	16.00	28.0	848.0	43.00
N:P	mol/L	43.23	77.59	61.95	89.8	1246.5	65.33
Chlorophyll	μg/L	3.40	14.90	6.50	16.2	360.0	23.00

**Table 4.** Percentage of lakes and reservoirs in different chlorophyll-based trophic states.

Source	Oligotrophic	Mesotrophic	Eutrophic	Hypereutrophic
URIWW	21.4	42.9	20.2	15.5
LAGOS-NE	9.7	37.6	26.1	26.7

**Table 5.** Summary of long-term water quality trends in the URIWW and LAGOS-NE data for temperature (Temp.) chlorophyll (Chl.), total nitrogen (TN), total phosphorus (TP), and the molar nitrogen-tophosphorus ratio (N:P).

Source	Temp.	Chl.	TN	TP	N:P
URIWW	++	++	0	0	0
LAGOS-NE		0	0	+	0

*Note:* The “++” indicates a positive trend, the “+” indicates a weak positive trend, and the “0” indicates no trend.
